# miR-302b-3p Promotes Self-Renewal Properties in Leukemia Inhibitory Factor-Withdrawn Embryonic Stem Cells

**DOI:** 10.22074/cellj.2018.4846

**Published:** 2017-12-24

**Authors:** Sharif Moradi, Thomas Braun, Hossein Baharvand

**Affiliations:** 1 Department of Stem Cells and Developmental Biology, Cell Science Research Center, Royan Institute for Stem Cell Biology and Technology, ACECR, Tehran, Iran; 2 Department of Developmental Biology, University of Science and Culture, Tehran, Iran; 3 Max-Planck Institute for Heart and Lung Research, Department of Cardiac Development and Remodelling, Bad Nauheim, Germany

**Keywords:** Differentiation, Embryonic Stem Cells, MicroRNA, miR-302, Self-Renewal

## Abstract

**Objective:**

Embryonic stem cells (ESCs) are regulated by a gene regulatory circuitry composed of transcription factors, signaling pathways, metabolic mediators, and non-coding RNAs (ncRNAs). MicroRNAs (miRNAs) are short ncRNAs which play crucial roles in ESCs. Here, we explored the impact of miR-302b-3p on ESC self-renewal in the absence of leukemia inhibitory factor (LIF).

**Materials and Methods:**

In this experimental study, ESCs were cultured in the presence of 15% fetal bovine serum (FBS) and induced to differentiate by LIF removal. miR-302b-3p overexpression was performed by transient transfection of mature miRNA mimics. Cell cycle profiling was done using propidium iodide (PI) staining followed by flow cytometry. miRNA expression was quantified using a miR-302b-3p-specific TaqMan assay. Data were analyzed using t test, and a P<0.05 was considered statistically significant.

**Results:**

We observed that miR-302b-3p promoted the viability of both wild-type and LIF-withdrawn ESCs. It also increased ESC clonogenicity and alkaline phosphatase (AP) activity. The defective cell cycling of LIF-deprived ESCs was completely rescued by miR-302b-3p delivery. Moreover, miR-302b-3p inhibited the increased cell death rate induced by LIF removal.

**Conclusion:**

miR-302b-3p, as a pluripotency-associated miRNA, promotes diverse features of ESC self-renewal in the absence of extrinsic LIF signals.

## Introduction

Embryonic stem cells (ESCs) are isolated from blastocyst-stage embryos, and display multi-lineagedifferentiation potential and the ability to self-renew indefinitely in culture (1-3). These two key characteristics make ESCs invaluable tools for basic and appliedresearch on organismal development, drug discovery, toxicological studies, and disease modeling (4, 5). Serum-containing media supplemented with the leukemia inhibitory factor (LIF) have been used to maintain ESCs in an undifferentiated state (1, 2). In fact, serum provides bone morphogenetic protein (BMP) signals which inhibit neurogenesis while LIF blocks ESC differentiation into mesendoderm as well as supports ESC clonogenicity (6).

Importantly, culture media which contain inhibitors of ESC differentiation have been found to maintain ESCs in a more robust manner (7, 8). For example, R2i is a recently developed ESC culture medium which exploits the ability of small-molecule chemicals to inhibit endogenousdifferentiation signals in ESCs, i.e. transforming growth factor-ß (TGF-ß) and extra-cellular regulated kinase (ERK) pathways, thereby providing ESCs with a so- called ground state of pluripotency which is much more resistant to differentiation (9). ESC behavior is governed by a network of transcription factors (TFs), signaling pathways, chromatin regulators, and regulatory non- coding RNAs (ncRNAs) (10-13). In this integrated gene regulatory network (GRN), microRNAs (miRNAs) play pivotal parts to sustain pluripotency and promote self- renewal capacity (11, 14).

miRNAs are ~22-nt long ncRNAs which regulate a wide range of transcripts at the post-transcriptional level, thereby controlling virtually all developmental pathways and biological processes (15-18). These small RNAs are dynamically expressed and play important roles in different cellular states including during stem cell differentiation and cell state transitions (15, 19). ESCs express a specific set of miRNAs, and exhibit major rearrangements in miRNA profiles upon exit from pluripotency (20). Moreover, miRNAs are differentially expressed and are functionally important over the course of somatic cell reprogramming to pluripotency-a process also known as induced pluripotent stem (iPS) cell generation (15). ESC behavior is orchestrated by a unique group of miRNAs, among which embryonic stem cell cycle-regulating (ESCC) miRNAs represent the most crucially important players (21, 22). ESCC miRNAs include some members of miR-17 family, miR-290~295 cluster, and miR-302~367 cluster (23). ESCC miRNAs have been shown to maintain ESC self-renewal in the presence of differentiation-inducing miRNAs (let-7 family) (24). However, it has remained uncharacterized whether ESCC miRNAs can promote diverse aspects of stem cell self- renewal in the absence of LIF, a situation which impairs the undifferentiated maintenance of ESCs in terms of cell cycling, clonogenicity, viability, and pluripotency gene expression. In this study, we sought to determine whether miR-302b-3p, as an ESCC miRNA belonging to miR-302~367 cluster, could restore normal self-renewal to LIF-withdrawn ESCs. We chose miR-302b-3p for functional analysis because i. It is an ESCC miRNAs (the most functionally important class of ESC miRNAs); and ii. It has been analyzed in the context of iPS cell generation and wild-type ESCs to some extent, and therefore we wanted to further investigate it in a new context (i.e. LIF withdrawal) which has not been previously analyzed. We observed that cell cycle defects of LIF-withdrawn ESCs were rescued by miR-302b-3p. In addition, we found that miR-302b-3p stimulated the viability of ESCs both in the presence and absence of LIF and inhibited the increased cell death induced by LIF removal. Overall, we report that miR302b- 3p is a potent driver of ESC self-renewal in the absence of differentiation-inhibiting extrinsic signals.

## Materials and Methods

### Cell culture

In this experimental study, mouse ESCs (9) were cultured on gelatin-coated tissue-culture plates (Sigma-Aldrich, USA) in Knockout™ DMEM (Invitrogen, USA) supplemented with 15% ES-qualified fetal bovine serum (HyClone, UK), 2 mM L-glutamine (Invitrogen, USA), 0.1 mM nonessential amino acids (Invitrogen, USA), 100 U/ml penicillin, 100 µg/ml streptomycin (Invitrogen, USA), 0.1 mM ß-mercaptoethanol (Sigma-Aldrich, USA), and 1000 U/ml mouse LIF (mLIF, Royan Biotech, Iran), and sub-cultured every second day. R2i cells were cultivated in N2B27 media consisting of Neurobasal^®^ medium and DMEM/F-12 (both from Invitrogen, USA) at a 1:1 ratio, 1% B27 supplement (Invitrogen, USA), 1% N2 supplement (Invitrogen, USA), 0.1 mM non-essential amino acids, 5 mg/ml BSA(Invitrogen, USA), 2 mM L-glutamine, 0.1 mM ß-mercaptoethanol, 100 U/ml penicillin, 100 µg/ml streptomycin, 1 µM PD0325901 (Stemgent, USA), 10 µM SB431542 (Sigma-Aldrich, USA), and 1000 U/ml mLIF. This work was approved by the Ethical/ Scientific Committee of Royan Institute (Approval code: Ec/93/1137).

### Small RNA transfection

ESCs were transfected with 100 nM of miR-302b-3p mimics (Dharmacon, miRIDIAN microRNA mimics, Thermo Fisher Scientific, USA) according to the vendor’s instructions. The scrambled small RNA control (Scr) or the miR-302b-3p mimics as well as the DharmaFECT1 transfection reagent (Dharmacon, Thermo Fisher Scientific, USA) were diluted in serum-free DMEM/F-12, mixed, and incubated for 20 minutes at room temperature. DharmaFECT1-small RNA complexes were added to the culture media in a drop-wise manner. Assays were performed with three biological replicates and the data are represented as the mean ± SEM.

### Alkaline phosphatase staining 

To analyze alkaline phosphatase (AP) activity, cells were rinsed with phosphate buffered saline (PBS), fixed with a solution of acetone, 37% formaldehyde, and citrate solution, washed with deionized water, and then stained using a Leukocyte Alkaline Phosphatase Kit (Sigma- Aldrich, USA) for 15 minutes at room temperature. Next, the cells were washed with, and stored in, PBS.

### Clonogenicity assay

ESCs (6.0×10^4^ cells/well of 12-well plates) were transfected with miR-302b-3p mimics 1 day after seeding. Three days post-transfection, cells were replated at 5.0×10^3^ cells/well on gelatinized 24-well plates. On day 5 after replating, AP staining was carried out, and undifferentiated (AP-positive) and differentiated (AP-negative) ESC colonies were counted to determine cloning efficiency. 

### Quantitative reverse transcription-polymerase chain reaction 

Total RNAwas isolated using miRVana™ miRNAIsolation Kit (Invitrogen, USA) or miRNeasy Micro Kit (Qiagen, Germany) following the vendor’s instructions. To detect mRNAs using quantitative reverse transcription-polymerase chain reaction (qRT-PCR), 2 µl cDNA (12.5 ng) was used in a 10 µl PCR reaction using the Power SYBR^®^ Green PCR Master Mix (Life Technologies, USA) and gene-specific primers (Table 1). Expression of mRNAs was normalized against *Gapdh* using the ΔΔCt method.

For detection and quantitation of miR-302b-3p using qRT-PCR, cDNA was synthesized from 20 ng of total RNA using miR-302b-3p-specific TaqMan miRNA RT primer and amplified using a miR-302b-3p-specific TaqMan^®^ assay (Applied Biosystems, USA). snoRNA202 was used as an internal normalization control. Reactions were run on a StepOnePlus™ machine (Applied Biosystems, USA) in triplicates and data were analyzed using the ΔΔCt method. 

**Table 1:** Primer sequences used for quantitative reverse transcriptionpolymerase chain reaction


Gene	Primer sequences (5ˊ-3ˊ)

*Gapdh *	F: GACTTCAACAGCAACTCCCAC
	R: TCCACCACCCTGTTGCTGTA
*Esrrb*	F: AGGCTCTCATTTGGGCCTAGC
	R: ATCCTTGCCTGCCACCTGTT
*Rex1 *	F: TAGCCGCCTAGATTTCCACT
	R: GTCCATTTCTCTAATGCCCAC
*Dppa3 *	F: CTTTGTTGTCGGTGCTGAAA
	R: GTCCCGTTCAAACTCATTTCC
*Cdh1 *	F: GCTGGACCGAGAGAGTTAC
	R: GGCACTTGACCCTGATACG


### Cell cycle analysis

ESCs were seeded at 2.0×10^5^ cells/well in 6-well plates 1 day prior to miR-302b-3p delivery, harvested on day 3 post-transfection, rinsed with PBS, fixed with ice-cold 70% ethanol, and then incubated at -20°C for at least 2 hours before washing with ice-cold PBS. The cells were resuspended in propidium iodide (PI)/RNase Staining Buffer (12.5 µg/ml PI and 100 µg/ml RNase) and incubated at room temperature for 15-30 minutes in the dark. Flow cytometry was carried out using a BD LSR II flow cytometer (BD Biosciences, USA) and the data analysis was done with BD FACSDiva (BD Biosciences, USA). 

### Cell viability assays 

#### Live/dead viability assay

Cells were incubated with the reagent [0.1 µM ethidium homodimer-1 and 0.1 µM calcein acetoxymethyl ester (calcein AM) in PBS] from the Live/Dead^®^ Viability/ Cytotoxicity Kit for Mammalian Cells (Molecular Probes, USA) at room temperature for 30-60 minutes. The cells were then washed with PBS and visualized under fluorescence microscope (Olympus, IX71, Japan). 

#### MTS viability assay 

After removal of medium, the MTS reagent (Promega, USA) was directly added to the wells in 96-well plates, and the cells were then maintained in a 37°C incubator for 1-3 hours. Cell viability measurements were performed by determining absorbance at 495 nm on a Multiskan MCC microplate reader (Thermo Fisher Scientific, USA).

### miRNA target prediction and gene ontology analysis 

TargetScan [www.targetscan.org (25)], miRanda [http://www.microrna.org/ (26)], and miRWalk [http://zmf.umm.uni-heidelberg.de/apps/zmf/mirwalk2/ (27)] tools were used to predict the potential mRNA targets of miR-302b-3p. The predicted targets were subjected to gene ontology (GO) Biological Process and Wikipathways analyses using miRWalk and Enrichr [http://amp.pharm.mssm.edu/Enrichr/ (28)]. Only GO terms with a P<0.05 were considered statistically significant and represented.

### Statistical analysis

Data are shown as means ± SEM. Student’s t test was used to analyze differences, and a P<0.05 was considered statistically significant. GraphPad PRISMTM software was used for data analysis. 

## Results

### miR-302b-3p promotes embryonic stem cell viability 

**Fig.1:**
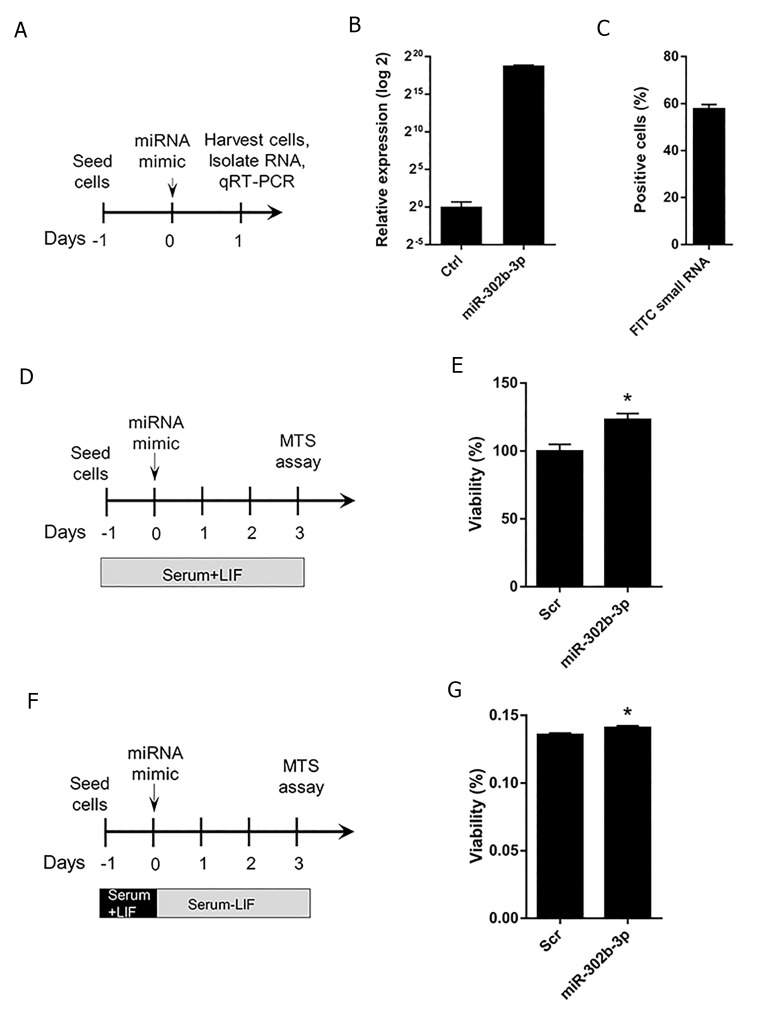
miR-302b-3p promotes ESC viability. A. Procedure of miR-302b-3p mimic delivery into MEFs, B. qRT-PCR analysis of miR-302b-3p expression levelfollowing miRNA transient transfection. Data areshown asmean ± SEM, n=3, C. The efficiency of FITC-small RNA transfection into ESCs asdetermined byflow cytometry 24 hours after transfection. Data areshown asmean ± SEM, n=3, D. Procedure of miR-302b-3p delivery into wild-type ESCs (serum+LIF) for viability assessment, E. MTS assay of wild-type ESCs 3 days after treatment with miR-302b-3p. Data areshown asmean ± SEM, n=3 (*; P<0.05), F. Procedure of miR-302b-3p transfection into LIF-withdrawn ESCs for viability assessment, and G. MTS assay of LIF-withdrawn ESCs 3 days after transfectionwith miR-302b-3p. Data areshown asmean ± SEM, n=3 (*; P<0.05). ESC; Embryonic stem cells, MEFs; Mouse embryonic fibroblasts, qRT-PCR; Quantitative reverse transcription-polymerase chain reaction, and LIF; Leukemiainhibitory factor.

First, we wanted to examine whether miR-302b-3p could promote the viability of wild-type ESCs. To this end, we confirmed that our miRNA delivery system was efficient enough for miRNA overexpression. Mouse embryonic fibroblasts (MEFs), which do not express this miRNA, were seeded 1 day prior to miRNA treatment and harvested for qRT-PCR analysis 1 day post- treatment (Fig .1A). Our results showed that compared to non-transfected control cells, MEFs transfected with miR-302b-3p mimics highly expressed the mature miRNA mimics (Fig .1B), indicating that our delivery system was highly efficient. In addition, to assess the efficiency of small RNA transfection into ESCs, we used FITC-conjugated small RNAs for transient transfection of ESCs. Our data using flow cytometry revealed that 24 hours post transfection, almost 60% of ESCs could uptake the FITC-conjugated small RNAs (Fig .1C). 

Next, we treated wild-type ESCs with miR-302b3p mimics and performed MTS assay 3 days posttransfection (Fig .1D). Our data indicated that ESCs treated with miR-302b-3p exhibited significantly enhanced viability compared to the Scr control, as manifested by MTS assay (Fig .1E). Therefore, miR302b- 3p mimics promote the viability of wild-type ESCs. In addition, LIF-withdrawn ESCs were treated with miR-302b-3p mimics (Fig .1F) and exhibited an improved viability 3 days post-transfection (Fig .1G). Overall, we conclude that miR-302b-3p increases the viability of both serum+LIF and serum-LIF ESCs.

### Embryonic stem cell clonogenicity is enhanced by miR-302b-3p 

**Fig.2:**
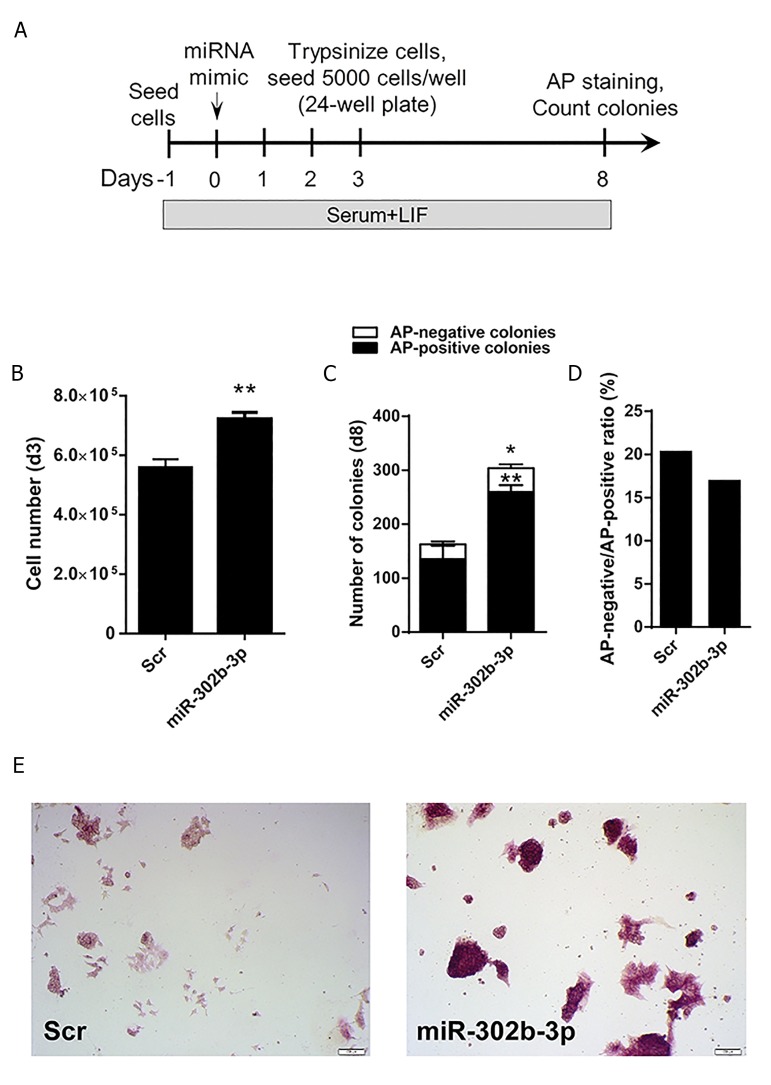
Effect of miR-302b-3p on ESC cloning efficiency and AP activity. A. Procedure of clonogenicity analysis of ESCs after transfection with miR-302b3p, B. Analysis of cell number 3 days after ESC treatment with miR-302b-3p. Data are shown as mean ± SEM, n=3 (*; P=0.0092), C. Cloning efficiency of ESCs 8 days after ESC treatment with miR-302b-3p mimics. Data are shown as mean ± SEM, n=3 (*; P<0.05 and **; P=0.0015), D. Ratio of AP-positive ESC colonies to AP-negative colonies 8 days after treatment with miR-302b-3p mimics, and E. Analysis of AP activity of ESCs treated with miR-302b-3p on day 8 post-transfection. ESC; Embryonic stem cells and AP; Alkaline phosphatase.

We next asked if miR-302b-3p could regulate the colony-forming efficiency of ESCs. To this end, ESCs were seeded 1 day prior to miRNA transfection, reseeded 3 days post-transfection, and subjected to AP staining 5 days after re-seeding (Fig .2A). We found that the number of ESCs was significantly increased 3 days after treatment with miR-302b-3p compared to Scr (Fig .2B). Moreover, we observed that on day 8, there was a considerably larger number of AP-positive ESC colonies after miR-302b-3p transfection, suggesting that miR-302b-3p promoted the clonogenicity of ESCs. Of note, the number of AP-negative colonies was higher in miR-302b-3p-treated cells compared to Scr-treated cells (Fig .2C), which might imply that miR-302b-3p also stimulates exit from pluripotency. However, we observed that the ratio of AP-positiveto AP-negative colonies was higher in Scr than miR302b- 3p-treatment group (Fig .2D), suggesting that miR-302b-3p limited the silencing of ESC self-renewal program. Furthermore, we noticed that ESCs treated with miR-302b-3p mimics exhibited a remarkably higher AP activity, as evidenced by the enhanced AP staining intensity in miR-302b-3p-trasfected cells compared to the Scr control (Fig .2E). These data indicate that miR-302b-3p is a potent driver of ESC self-renewal. 

### miR-302b-3p restores normal cell cycling to leukemia inhibitory factor-withdrawn embryonic stem cells

ESCs have a unique cell division cycle which is tightly regulated by numerous pluripotency-associated factors including miRNAs (29-31). In fact, ESCC miRNAs control key aspects of ESC cycling program which in turn positively affects their pluripotency and unlimited proliferation in culture. miR-302b-3p is a member of ESCC miRNAs which play important roles in the cell cycle fine-tuning of wild-type ESCs (21). However, whether miR-302b-3p (and therefore ESCC miRNAs) could promote ESC cycling in the face of LIF withdrawal is not known. LIF removal is known to trigger ESCs to exit from pluripotency by lengthening their G1 phase. 

**Fig.3:**
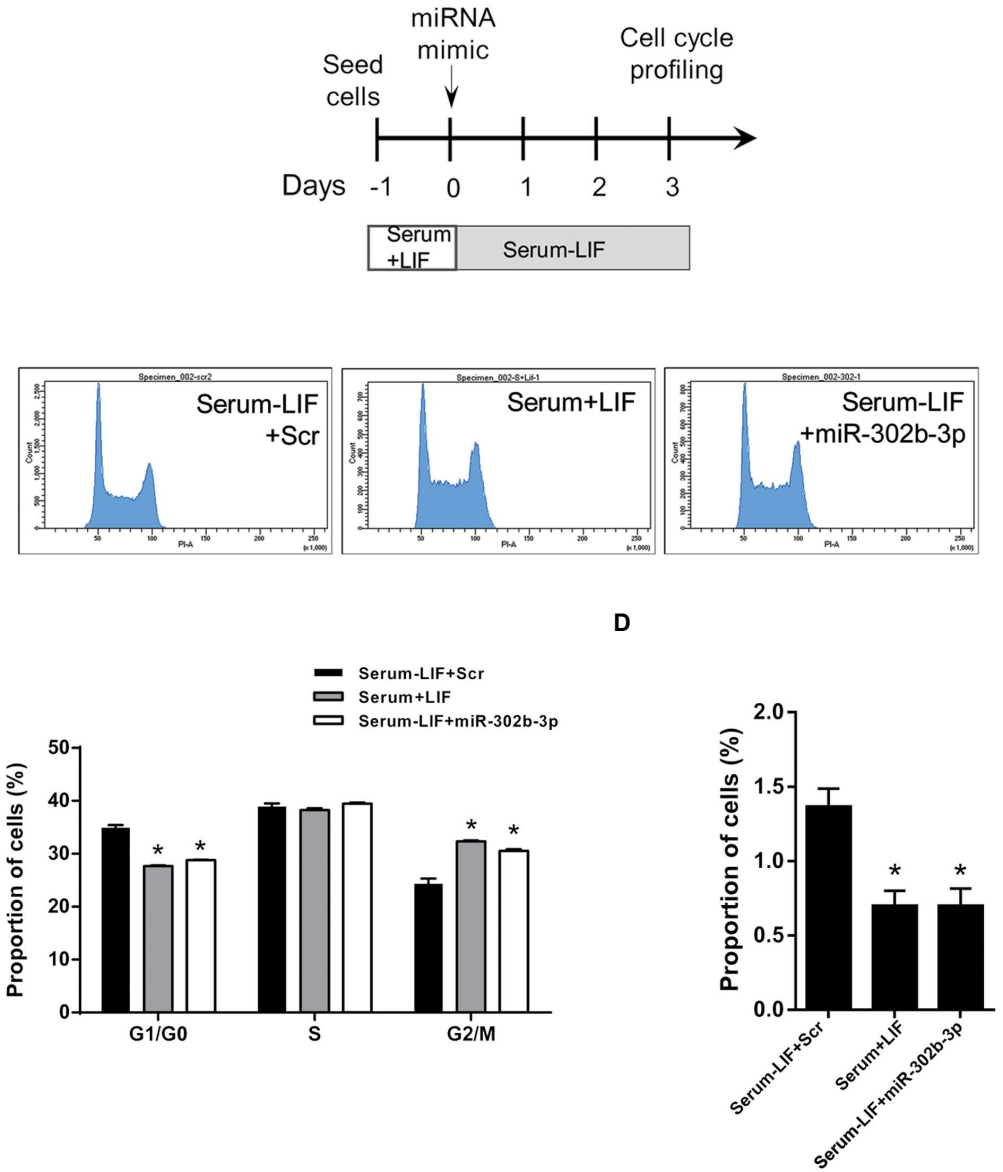
Cell cycle profiling of LIF-withdrawn ESCs treated with miR-302b-3p. A. Procedure of cell cycle analysis of LIF-deprived ESCs after transfection with miR-302b-3p, B. Histograms of cell cycle profiles of wild-type ESCs aswell asLIF-withdrawn ESCs in the presence or absence of miR-302b-3p, C. Barplotshowing the cell cycle status of LIF-withdrawn ESCs in the presence or absence of miR-302b-3p using PI staining followed by flow cytometry 3 days post- transfection. Data areshown asmean ± SEM, n=3 (*; P<0.05), and D. Percentage of wild-type ESCs and LIF-withdrawn ESCs in the presence or absenceof miR-302b-3p in sub-G1 phase 3 days post-transfection determined using PI staining followed by flow cytometry. Data arerepresented asmean ± SEM, n=3 (*; P<0.05). LIF; Leukemia inhibitory factor, ESC; Embryonic stem cells, and PI; Propidium iodide.

We wanted to examine whether the introduction of miR-302b-3p could restore normal cell cycling to LIF- deprived ESCs which display a defective cell cycle profile. To test this hypothesis, we removed LIF from the ESC culture media and concomitantly added miR302b- 3p. Three days following treatment with miR-302b3p mimics, cell cycle was assessed by flow cytometry following PI staining (Fig .3A). Our data indicated that LIF removal extended G1 phase in ESCs compared to ESCs cultured under serum+LIF condition, which is in agreement with previous findings (32, 33). Importantly, we observed that miR-302b-3p significantly shortened the extended G1 phase of LIF-deprived ESCs to levels comparable to serum+LIF cells (Fig .3B, C). This result indicated that miR-302b-3p restored normal cell cycling to LIF-withdrawn ESCs. Moreover, we observed that LIF removal triggered a significant increase in cell death rate. However, miR-302b-3p treatment could compensate for the absence of LIF by significantly reducing cell death compared to the Scr control (Fig .3D). Taken together, the defective cell cycle and enhanced cell death of LIF- deprived ESCs are rescued by miR-302b-3p.

### miR-302b-3p stimulates viability of ground-state embryonic stem cells

**Fig.4:**
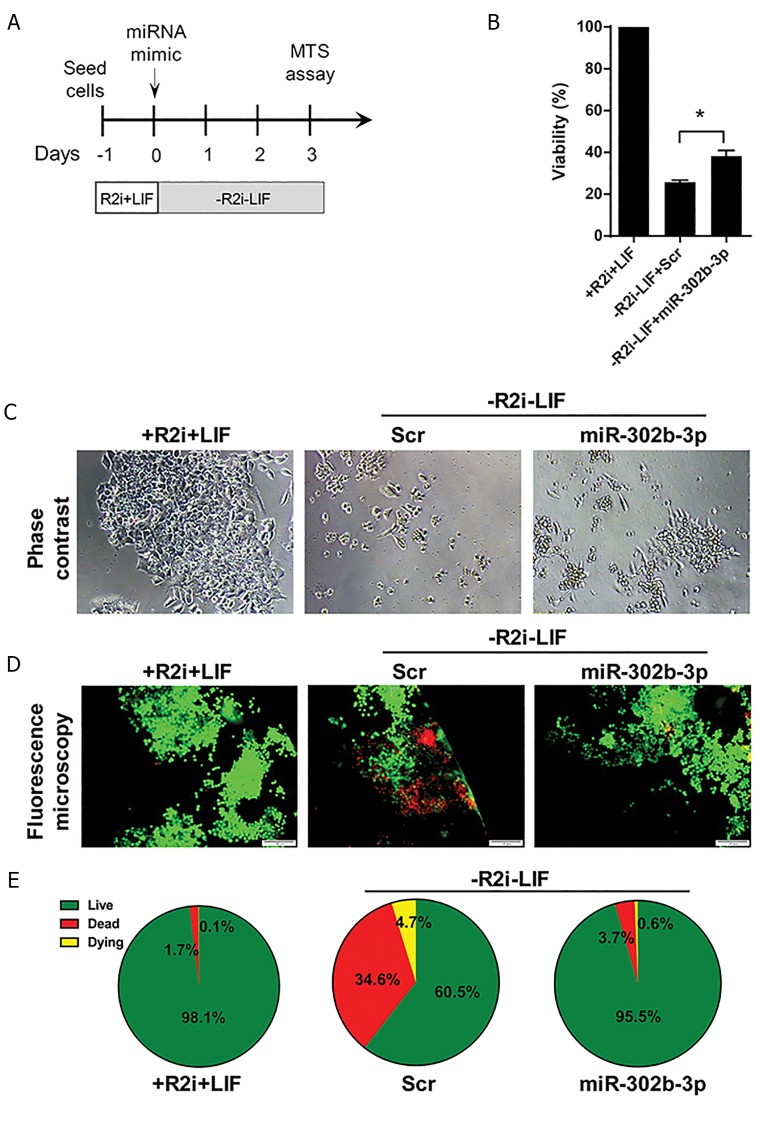
miR-302b-3p promotes the viability, and inhibits the death, of LIF-withdrawn ESCs. A. Procedure of ESC treatment with miR-302b-3p for viability assessment and cell death analysis, B. Barplot showing the MTS assay of LIF-deprived ESCs 3 days after transfection with miR-302b-3p. Data are represented as mean ± SEM, n=3 (*; P<0.05), C. Phase contrast image of R2i/LIF ESCs, R2i/LIF-withdrawn ESCs, and R2i/LIF-withdrawn ESCs treated with miR-302b-3p, D. Live/dead immunofluorescence staining of R2i/LIF-withdrawn ESCs 3 days after miR-302b-3p transfection (scale bar: 100 µm), and E. Quantification of the live (green), dead (red), and dying (yellow) cells shown in (D) using Image J. LIF; Leukemia inhibitory factor and ESC; Embryonic stem cells.

Since miR-302b-3p promoted the viability of wild-type ESCs as well as the normal cell cycling in the absence of LIF, we then examined whether miR-302b-3p could positively influence the viability of ESCs upon LIF withdrawal. To provide a proper model for this analysis, we cultured the cells in R2i+LIF, a culture condition which consists of LIF plus small-molecule inhibitors of FGF-ERK and TGF-ß signaling pathways and promotes the ground state properties in ESCs (9). We then acutely removed R2i chemicals as well as LIF from the culture which led to a significant reduction in cell viability as well as a marked increase in the rate of cell death (Fig .4A). Our results indicated that 3 days after R2i/LIF removal, the viability of R2i/LIF-withdrawn ESCs was markedly diminished compared to ESCs grown in R2i+LIF condition. 

Interestingly, we observed that miR-302b-3p could significantly enhance the viability of R2i/LIF-deprived ESCs compared to the Scr control and partially rescue them (Fig .4B). R2i/LIF-withdrawn ESCs treated with miR-302b-3p were also found to have larger colonies (and therefore larger number of cells) compared to the Scr control (Fig .4C), indicating that miR-302b-3p inhibited the increased cell death rate induced by the removal of LIF (and R2i). To confirm the observation that miR-302b-3p stimulates the viability of R2i/LIF-withdrawn ESCs, we analyzed the degree of cell death following R2i/LIF withdrawal using Live/Dead Staining Kit. Our results revealed that 3 days after addition of miR-302b-3p mimics (at the time of R2i/LIF removal), there was a remarkably larger number of green (live) cells compared to the Scr control which displayed a much larger number of red (dead) and yellow (dying) cells (Fig .4D, Fig 4E). These collective data indicated that miR-302b-3p provision could partially compensate for the lack of R2i chemicals and LIF in the maintenance of ESC self-renewal. 

### miR-302b-3p potentially targets multiple pathways to promote embryonic stem cells self-renewal 

**Fig.5:**
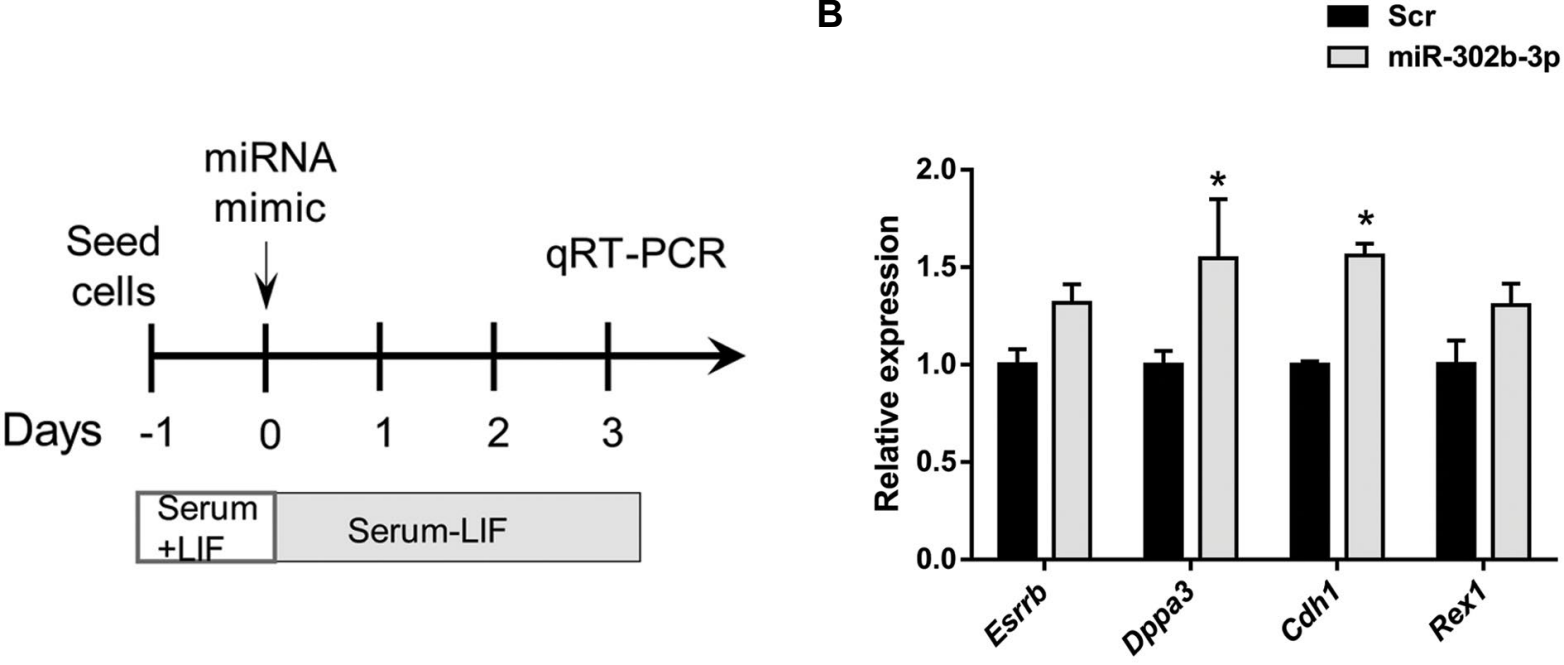
qRT-PCR analysis of ESC-associated gene expression 3 days following miR-302b-3p transfection into LIF-withdrawn ESCs. Data are shown as mean ± SEM, n=3 (*; P<0.05). A. Procedure of ESC treatment with miR-302b-3p mimics for pluripotency gene expression analysis and B. Barplot indicating the expression pattern of pluripotency-associated genes 3 days post-transfection. Data are represented as mean ± SEM, n=3 (*; P<0.05). qRT-PCR; Quantitative reverse transcription-polymerase chain reaction, ESC; Embryonic stem cells, and LIF; Leukemia inhibitory factor.

miRNAs are known to regulate many cellular processes in different contexts. Some miRNAs appear to exert their cellular effects mainly through inhibiting one or a few number of transcripts whereas others fine-tune numerous transcripts to induce a certain cellular phenotype (34). To examine if miR-302b-3p treatment promote the expression of typical genes associated with ESC pluripotency, we removed LIF from ESC culture media and concomitantly treated them with miR-302b-3p (Fig .5A). Our data showed that 3 days after miRNA transfection, LIF-withdrawn ESCs exhibited a stimulation of pluripotency gene expression (Fig .5B), which suggests that miR-302b3p contributes to the maintenance of LIF-withdrawn ESCs by promoting ESC-specific gene expression.

**Fig.6:**
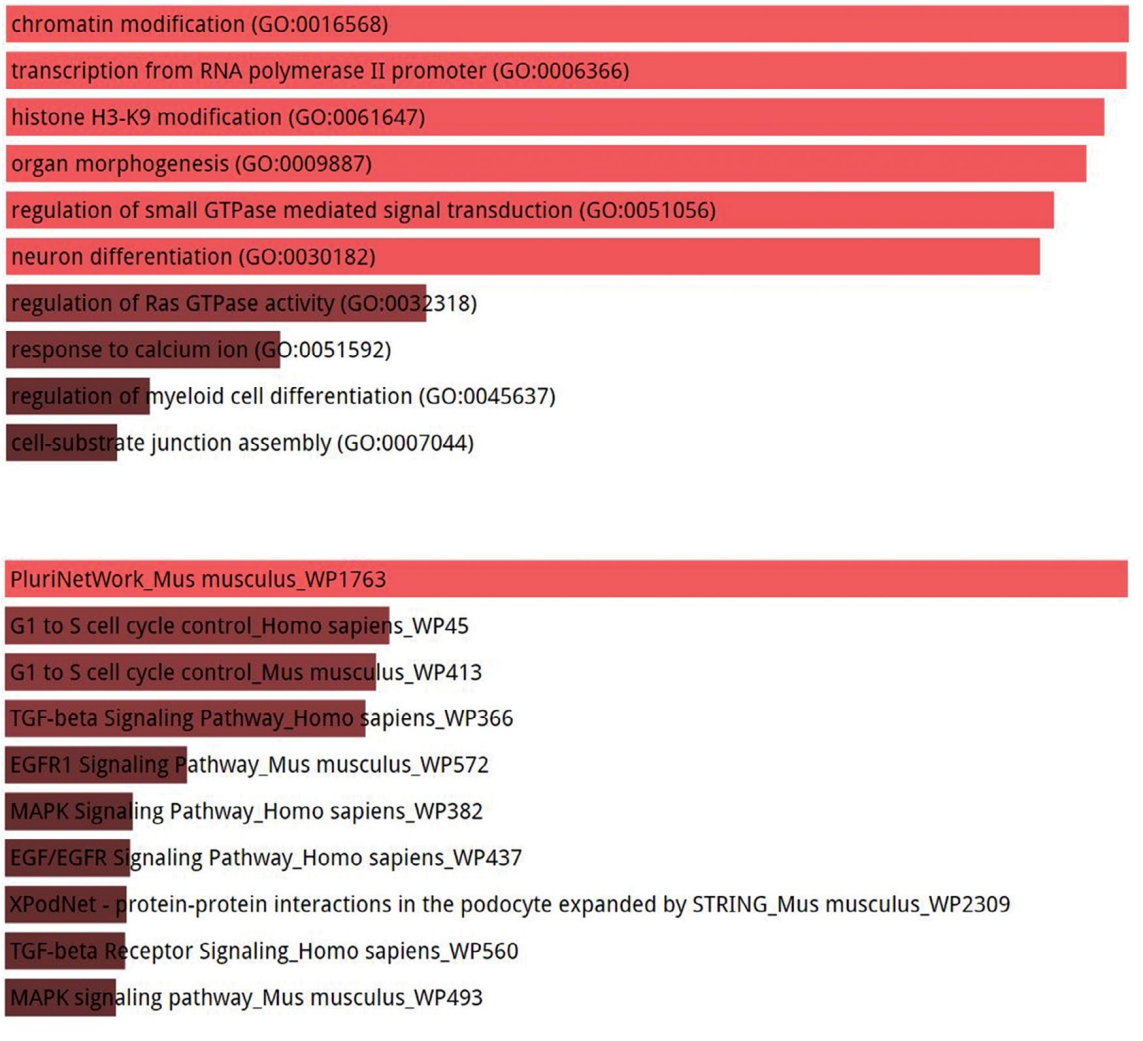
Biological pathways potentially regulated by miR-302b-3p. A. Enrichr-based GO Biological Process analysis of miR-302b-3p targets predicted by TargetScan and B. Enrichr-based Wikipathways analysis of miR-302b-3p targets predicted by TargetScan.

Next, to gain insight into the putative biological pathways regulated by miR-302b-3p in ESCs, we used the TargetScan algorithm to obtain predicted targets of miR-302b-3p. Based on family seed sequence and target site conservation, TargetScan provided predicted targets of miR-302 seed family (Table S1) (See supplementary Online Information at www. celljournal.org) which we used for GO analysis. Our GO Biological Process analysis of miR-302b-3p predicted targets using Enrichr suggested that it might control chromatin status as well as important pathways associated with differentiation including organ morphogenesis (Fig .6A). Moreover, Wikipathways feature of Enricher suggested that typical signaling pathways associated with ESC differentiation [FGFERK-(MAPK) and TGF-ß pathways (7, 9)] are potentially targeted by miR-302b-3p. ESCs have a distinct cell cycle and, interestingly, miR-302b-3p was predicted to regulate cell cycle progression (Fig .6B).

To evaluate the results obtained by Enrichr, we simultaneously used three miRNA target prediction tools (miRWalk, miRanda, and TargetScan). Our GO Biological Process analysis using miRWalk (Table 2) suggested that different differentiation pathways, chromatin structure, cell cycle, and TGF-ß signaling are potentially regulated by miR-302b-3p, thereby confirming the Enrichr results. Additionally, miR-302b 3p was predicted to inhibit epithelial to mesenchymal transition (EMT) as well as apoptosis, which might contribute to the maintenance of undifferentiated ESCs. Taken together, miR-302b-3p appears to control diverse cellular pathways to promote ESC self-renewal in the absence and/or presence of LIF.

**Table 2:** miRWalk Biological Process analysis of miR-302b-3p predicted targets


Pathway name	P value

Apoptotic process	5.98E-06
Chromatin modification	2.26E-05
Hemopoiesis	0.000146469
Cell cycle	0.001613578
Embryonic organ development	0.001729511
Axonogenesis	0.00241029
Neuron projection development	0.006984727
TGF-β signaling pathway	0.009703597
Muscle cell differentiation	0.008556945
B cell differentiation	0.011624291
Post embryonic development	0.012442256
Vasculature development	0.013997516
Forebrain morphogenesis	0.016093928
Spleen development	0.02050384
Endoderm development	0.030824682
Neuron differentiation	0.031794463
Cell fate commitment	0.03220361
Organ morphogenesis	0.038932781
Astrocyte differentiation	0.045223546
Epithelial to mesenchymal transition	0.044181992


## Discussion

In the present study, we investigated the functional significance of miR-302b-3p as an ESCC miRNA in ESCs. We found that miR-302b-3p not only promoted ESC viability in wild-type ESCs, but also enhanced the cellular viability of LIF-withdrawn ESCs. It also increased the number of undifferentiated ESC colonies at the expense of differentiated ones, and stimulated AP activity. miR-302b-3p inhibited the increased cell death rate upon LIF withdrawal and provided LIF- deprived ESCs with normal cell cycling typical of wild-type ESCs.

The observation that miR-302b-3p rescues LIF- withdrawn ESCs might be due to the ability of miR-302b-3p to inhibit multiple ESC-impairing pathways that become activated upon LIF removal. LIF is known to sustain ESC self-renewal through activating JAK-STAT3 signaling pathway and to inhibit differentiation in ESCs (6). Our bioinformatics analysis suggested that miR-302b-3p might contribute to the maintenance of LIF-withdrawn ESCs partly by inhibition of differentiation. Consistent with our GO analysis of miR-302b-3p predicted targets, miR302 seed family has been experimentally validated to inhibit neuroectodermal differentiation (35, 36). TGF-ßand MAPK pathways are also predicted to be inhibited by miR-302b-3p. These two pathways are well-known differentiation-affiliated pathways, and their dual inhibition has been reported to promote the establishment and maintenance of ground state pluripotency in ESCs, a culture condition developed recently which is called R2i (9). In principle, the observation that R2i/LIF ESCs are partially rescued by miR-302b-3p might be due to the miR-302b-3p based inhibition of these two signaling pathways that are normally inhibited in R2i culture. The point that differentiation pathways might be inhibited by miR-302b-3p in LIF-withdrawn ESCs can be best explained by the fact that the miR-302~367 cluster efficiently promotes the de-differentiation of somatic cells into iPS cells in the presence and/or absence of reprogramming TFs (15). 

It is known that LIF removal triggers exit from the typical cell cycle of ESCs (i.e. prolongs G1 phase) (32, 33). We observed that miR-302b-3p completely inhibited the G1 phase extension induced by LIF removal. Indeed, miR-302b-3p (and other ESCC miRNAs) has been observed to inhibit the G1 restriction point by suppressing retinoblastoma (Rb) family of proteins, thereby protecting ESCs from exiting the cell division cycle (37). 

LIF removal and therefore ESC differentiation accompanies a process of EMT during which epithelial ESCs turn into a mesenchymal cell state to start differentiating (38, 39). miR-302 family of miRNAs are predicted and also reported by Guo et al. (40) and Liao et al. (41) to suppress EMT and apoptosis which might also explain why miR-302b-3p markedly inhibits cell death induced by R2i/LIF withdrawal. miR-302b3p might also exert some of its diverse effects through the regulation of chromatin status, as it gives rise to chromatin opening and ESC-type gene expression patterns during somatic cell reprogramming (15, 42). We conclude that ESCC miRNAs are integrated into a robust GRN in ESCs to promote ESC survival and undifferentiated self-renewal by modulating cell cycle, differentiation, and cell death. It remains to be experimentally determined how miR-302b-3p, and probably other ESCC miRNAs, are able to stimulate pluripotency maintenance in the absence of extrinsic LIF signals. 

## Conclusion

ESCC miRNAs represent the most functionally important class of miRNAs in ESCs. They are reportedly able to oppose differentiation-affiliated miRNAs (let-7 family) in ESCs. Serum-grown ESCs depend on extrinsic LIF signals to maintain self-renewal, and LIF-deprived ESCs are not able to sustain their undifferentiated state. In the present study, we examined if miR-302b-3p, as an ESCC miRNA, was able to restore self-renewal to LIF-withdrawn ESCs. Our data showed for the first time that miR-302b-3p could promote cell cycling, viability, pluripotency gene expression, AP activity, and clonogenicity as well as decrease cell death in LIF- withdrawn ESCs. We therefore conclude that ESCC miRNAs promote diverse aspects of ESC self-renewal in the absence of LIF.

## Supplementary PDF


