# Supplementary material for: miR-302b-3p Promotes Self-Renewal Properties in Leukemia Inhibitory Factor-Withdrawn Embryonic Stem Cells
Source: Cell J. 2017 Dec 24;20(1):61–72. doi: 10.22074/cellj.2018.4846 (PMC5761148; doi:10.22074/cellj.2018.4846)
Supplement: Supplementary file 1 [file Cell-J-20-61-s01.pdf]

CELL JOURNAL (Yakhteh)

www.celljournal.org

Supplementary Information for

miR-302b-3p Promotes Self-Renewal Properties in Leukemia Inhibitory Factor-Withdrawn Embryonic Stem Cells

Sharif Moradi, Ph.D.[1, 2], Thomas Braun, M.D., Ph.D.[3]*, Hossein Baharvand, Ph.D.[1, 2]*

1. Department of Stem Cells and Developmental Biology, Cell Science Research Center, Royan Institute for Stem Cell Biology and Technology, ACECR, Tehran, Iran
2. Department of Developmental Biology, University of Science and Culture, Tehran, Iran
3. Max-Planck Institute for Heart and Lung Research, Department of Cardiac Development and Remodelling, Bad Nauheim, Germany

***Corresponding Addresses: P.O.Box: 16635-148, Department of Stem Cells and Developmental Biology, Cell Science Research Center, Royan Institute for Stem Cell Biology and Technology, ACECR, Tehran, Iran**
Max-Planck-Institute for Heart and Lung Research, Ludwigstr. 43, 61231 Bad Nauheim, Germany
Emails: Baharvand@Royaninstitute.org, Thomas.braun@mpi-bn.mpg.de

Table S1: List of potential miR-302b-3p targets predicted by TargetScan

Human ortholog of target gene							
ZNF800	*TOX*	*MAP3K2*	*BCL6*	*HNRNPUL2*	*OXR1*	*ZFPM2*	*HUWE1*
TGFBR2	*SUV39H1*	*COL17A1*	*EPHA2*	*MED12L*	*ZNF597*	*KLHL28*	*ZBTB11*
ZBTB5	*LRIT1*	*MFN2*	*ZNF2*	*RSRC2*	*TBC1D8B*	*C2CD2*	*SHC4*
DDHD1	*PLAGL2*	*TXNIP*	*RSBN1*	*FOXI1*	*ANO6*	*PPP6C*	*BCL11B*
FGD4	*LEFTY2*	*EXPH5*	*IL28RA*	*ST3GAL1*	*ASH1L*	*ZBTB44*	*NEK9*
FBXL3	*ZNF697*	*LEFTY1*	*CXADR*	*SLITRK3*	*PFKP*	*BCL11A*	*ARHGAP29*
SYAP1	*RASAL2*	*OLIG2*	*SLC25A40*	*ATAD2*	*KLF3*	*ZBTB9*	*C1orf43*
LATS2	*EDNRB*	*FZD3*	*SYDE1*	*MNT*	*UNK*	*YTHDF3*	*TSHZ3*
ANKRD32	*DBR1*	*ITGB8*	*DPP8*	*SENP1*	*TMX1*	*CYBB*	*PRDM16*
ELAVL2	*ZNRF3*	*ARHGEF17*	*KPNA2*	*ACBD5*	*NR4A2*	*MINK1*	*FBXO11*
HIF1AN	*HIPK3*	*RASSF2*	*FBXL4*	*RNF6*	*C3orf18*	*APP*	*TSHZ2*
PARP8	*SYNC*	*PBX3*	*VSX1*	*PPP3R1*	*HCFC2*	*SLC16A12*	*MLL3*
FMR1	*ELK4*	*PAK7*	*PRR16*	*TFAP4*	*MARCH8*	*POLE3*	*ESR1*
IRF2BP2	*MLLT6*	*REEP3*	*SAR1B*	*VLDLR*	*CROT*	*ADAMTS18*	*BAMBI*
PHC3	*TRIM36*	*ASF1B*	*ARID4A*	*CDK2AP2*	*PTGDR*	*SS18L1*	*FAM134C*
PCGF5	*PRDM4*	*RGMA*	*C10orf140*	*MKNK2*	*RBMS2*	*FOXL2*	*GTDC1*
LHX6	*WDR37*	*FGF9*	*REST*	*DNAJA2*	*RBL1*	*C5orf22*	*GLDN*
LCORL	*TMEM123*	*LRP2*	*HMBOX1*	*CIRBP*	*UBE2Q2*	*TNRC18*	*ADAM9*
LCOR	*GDF11*	*IRF9*	*IKZF2*	*C1orf9*	*TAPT1*	*TIAM1*	*DIRC2*
ZKSCAN1	*CNOT6L*	*BTG1*	*MKRN1*	*C11orf95*	*DERL2*	*FOXJ3*	*FBXO10*
ASF1A	*FYCO1*	*PAX5*	*RRAGD*	*ECT2*	*C7orf43*	*RHOC*	*C15orf17*
AK2	*FOSB*	*ITFG1*	*LAMA3*	*TET1*	*ZFX*	*COMMD9*	*PAX6*
BRP44L	*WDR20*	*ISM2*	*ZFYVE26*	*E2F5*	*HLF*	*JRKL*	*CORO2B*
NR2C2	*RSBN1L*	*ASAP1*	*RPS6KA3*	*MTMR3*	*ZNFX1*	*ZNF148*	*SLC16A9*
GOLGA1	*CLIP4*	*KAT2B*	*SLAIN2*	*RYR2*	*STXBP5L*	*SDC1*	*TOX3*
ZNF367	*ARNT2*	*EPHA5*	*SMAD2*	*NR2C1*	*MYCN*	*LCLAT1*	*LHX8*
CDKN1A	*SASS6*	*USP24*	*RGMB*	*C16orf72*	*TMUB2*	*NETO1*	*TAOK2*
ARID4B	*TWF1*	*CELF2*	*TRIP11*	*SLC6A9*	*SPOP*	*RPS6KA1*	*NTN4*
CRIM1	*NFIB*	*ATF6B*	*INTS6*	*DCAF7*	*PGBD5*	*CCNJ*	*LUC7L2*
E2F2	*TIPARP*	*ITGB3*	*F3*	*NPAS3*	*POLQ*	*C11orf9*	*CNOT6*
UBE2B	*MCCD1*	*USP53*	*HS3ST4*	*POLK*	*GPR146*	*TBC1D20*	*SIK1*
RAB22A	*MBD2*	*SSR1*	*UNC80*	*PIP4K2A*	*KIAA0226*	*MEF2C*	*RTN1*
PRDM8	*ACSL6*	*A2LD1*	*USP42*	*TMCC1*	*NKX3-2*	*CRTC2*	*DMTF1*
TMEM170B	*CALM1*	*C11orf30*	*SRSF12*	*NR4A3*	*PCDH7*	*POU6F1*	*ACTR8*
PTCHD1	*FAM57A*	*MTUS1*	*CFL2*	*PLEKHA3*	*PHLPP2*	*TLE4*	*YOD1*
DOCK2	*CYP26B1*	*E2F7*	*KIAA1737*	*IPO7*	*ST8SIA2*	*TP53INP1*	*FGD5*
KIAA1549	*RALGDS*	*DENND5B*	*RB1CC1*	*UBE2W*	*SLC22A23*	*TNFAIP1*	*PTPN21*
SETBP1	*PRRG1*	*GPR6*	*RAB11FIP5*	*ARL4C*	*ERBB4*	*SMARCC2*	*ASXL2*
WDR1	*NFYA*	*BCAP29*	*IRF2*	*ARHGEF10*	*ABCA1*	*LEF1*	*MRPS25*
NCOA7	*ZNF22*	*SCN5A*	*HOXB13*	*DUSP2*	*RELL1*	*MAL2*	*MMP24*

Table S1: Continued

Human ortholog of target gene							
QSER1	*SLC40A1*	*CUX1*	*ORMDL3*	*CHN2*	*TCF20*	*KDM2A*	*ZDHHC9*
IRAK2	*DRD1*	*KCNMA1*	*FOXJ2*	*JOSD1*	*FAM78A*	*SPRED1*	*YPEL2*
TANC2	*ANKRD13C*	*RDBP*	*FNDC3A*	*TESK2*	*KLF13*	*KDM2A*	*GPC6*
KIAA1522	*INO80*	*GPR137C*	*CCND2*	*UBE3A*	*TRHDE*	*SPRED1*	*ACPL2*
FOXF2	*TNKS2*	*MFAP3L*	*DAZAP2*	*RAD18*	*MAP1B*	*APBB2*	*SIRPA*
NECAP1	*OSTM1*	*RAB5C*	*ZNF385A*	*PHF6*	*ANKRD54*	*AEBP2*	*SCRT2*
AAK1	*ERI1*	*HNRNPUL1*	*C19orf43*	*NRARP*	*PPP1R9A*	*PLXNA1*	*FAM102A*
RHOV	*RGL1*	*C10orf12*	*AKT1*	*HIVEP2*	*THSD7A*	*TNS1*	*POU2F1*
TRIM8	*LMO3*	*ZBTB41*	*PCBP3*	*CAMTA2*	*TCEB3*	*ZRANB1*	*SYNRG*
TOB2	*NFATC3*	*RBM33*	*GFAP*	*INHBB*	*CAMTA1*	*IGF2BP1*	*FAM155B*
CUL3	*CASC4*	*SSX2IP*	*SIPA1L3*	*ARX*	*MLL*	*ZCCHC24*	*ST8SIA3*
MTF1	*ARHGEF18*	*WDR45*	*SNRK*	*SNX21*	*HDAC4*	*NUFIP2*	*PRRX1*
TARDBP	*ITGB4*	*KIF3B*	*PLAG1*	*ATXN1*	*TMEM64*	*FAM168B*	*BEND4*
TET3	*TSLP*	*PFN2*	*KLHL18*	*TP63*	*ZNF238*	*HIC2*	*ZMYND11*
EZH1	*RECK*	*FAM117A*	*TRIM2*	*PHACTR4*	*TP53INP2*	*PAK2*	*TNRC6B*
KIF26B	*EIF4B*	*DSN1*	*KIAA1267*	*EPHA7*	*PHYHIPL*	*SBNO1*	*AGAP2*
CRK	*MBNL2*	*MCL1*	*GLIS3*	*TBC1D30*	*KLF12*	*RICTOR*	*SBF1*
FAM73B	*ATXN7L1*	*SLC2A4*	*KPNA1*	*ZBTB47*	*ZBTB7A*	*AGPS*	*PTPRD*
MAP3K14	*RNF216*	*JAZF1*	*ANAPC16*	*ZBTB43*	*CYB561D1*	*PSD3*	*ZMYM2*
SRCIN1	*ACER3*	*ZDHHC8*	*ERO1LB*	*MARK2*	*FAM175B*	*INO80D*	*SYT9*
UBE2J1	*MYT1L*	*CC2D1A*	*TET2*	*ALDH1L2*	*PARVA*	*MBNL3*	*ACVR1C*
ZNF518A	*NFIA*	*DPYSL5*	*CPEB2*	*AKTIP*	*PPP1R10*	*KCNA1*	*PIK3R3*
NAPEPLD	*DPP3*	*SLC25A27*	*ZNF507*	*LARP4*	*IP6K1*	*NNAT*	*MAML1*
STK38L	*ZNF362*	*WFS1*	*EIF2C1*	*FRMD4A*	*FAM46C*	*LIF*	*SLC35F2*
LRP8	*ZNF512B*	*CCDC88A*	*MAP3K11*	*DCUN1D1*	*C5orf41*	*TBCEL*	*SLC24A2*
ZC3H12C	*C1orf173*	*PTP4A1*	*KREMEN1*	*GATAD2B*	*ZFP91*	*CREB5*	*RNF38*
MAP3K1	*PKN2*	*ATP2C1*	*TTC9*	*FBXO41*	*PPARGC1B*	*SNX30*	*PAN3*
ARHGEF7	*RORB*	*AKAP6*	*ATG7*	*ARHGAP24*	*BCL2L11*	*RORA*	*SEC62*
MAST3	*GNB5*	*HAUS6*	*CCND1*	*WEE1*	*GRHL2*	*ALX4*	*ANKRD13B*
ABHD3	*RAB6A*	*LMX1A*	*BAHD1*	*ZNF436*	*LYPD6*	*FEM1C*	*CDH6*
SNX5	*DGKE*	*TUSC2*	*METAP1*	*VEGFA*	*HIP1*	*RAB11FIP1*	*UBN2*
P2RX4	*CTSA*	*NR2F2*	*ANKRD52*	*OSBPL5*	*CADM2*	*RUNX1*	*HN1*
BRMS1L	*RAPGEFL1*	*DKK1*	*CDK6*	*UHRF1BP1*	*VPS53*	*ATP2B2*	*TRPS1*
ZNF213	*TOR1B*	*MSL1*	*HS2ST1*	*TAL1*	*RAD23B*	*TNRC6C*	*UBFD1*
MKLN1	*ALKBH5*	*ULK1*	*CDK2*	*GIGYF1*	*UBE2R2*	*USP46*	*CLIC4*
CDCA7	*FZD6*	*RABEP1*	*AGFG2*	*MBNL1*	*SP3*	*PLEKHM1*	
WDR48	*UBXN2B*	*MYLK*	*C20orf112*	*BNC2*	*PEAK1*	*DNAJC27*	
UNKL	*MEX3A*	*NEUROD4*	*CDK19*	*CERS6*	*RAB11A*	*FOXN4*	
SAMD12	*MICAL3*	*WDR26*	*DCP2*	*REPS2*	*MYBL1*	*OPCML*	
TRPV6	*MIER3*	*PURB*	*BLCAP*	*EIF5*	*ZFHX4*	*IQSEC1*	
